# Effect of α-tocopheryl succinate on the molecular damage induced by indomethacin in C6 glioma cells

**DOI:** 10.3892/etm.2014.2101

**Published:** 2014-12-03

**Authors:** MURAT PEKMEZ, EVREN ÖNAY-UÇAR, NAZLI ARDA

**Affiliations:** Department of Molecular Biology and Genetics, Faculty of Science, Istanbul University, Istanbul 34134, Turkey

**Keywords:** α-tocopheryl succinate, indomethacin, C6 Glioma, intracellular ROS level, protein carbonyls

## Abstract

Indomethacin is a member of the non-steroidal anti-inflammatory drug (NSAID) class, which has great potential for use in the treatment of glioma. However, it induces the generation of reactive oxygen species (ROS) and causes molecular damage while inducing its effects. Vitamin E is widely used in the complementary therapy of cancers. The main goal of the present study was to investigate the effects of α-tocopheryl succinate (α-TOS) against the oxidative damage induced by indomethacin in C6 glioma cells. Cells were treated with 10 μM α-TOS alone or in combination with 200 μM indomethacin for two days. The intracellular ROS level, molecular damage as revealed by lipid peroxidation and protein carbonyl formation, and the COX activity in C6 glioma cells were measured. Treatment of the cells with α-TOS and indomethacin, alone or in combination, caused the levels of ROS generation and protein damage to increase, but protected against lipid peroxidation and reduced COX activity.

## Introduction

In general, all chemotherapeutic agents are toxic for healthy cells, and lower the quality of life due to harmful side-effects. Furthermore, they induce cellular oxidative stress (1). Following cancer chemotherapy, DNA oxidation and lipid peroxidation levels have been observed to be markedly increased in cancer patients (2). One of the clinical approaches for reducing the oxidative stress and harmful side-effects in chemotherapy is to use antioxidant vitamins and ROS scavengers such as vitamins A, C and E (2–5). Vitamin E derivatives, such as tocopherols (α, β, γ and δ) and tocotrienols (α, β, γ and δ) protect the cell membrane against oxidative stress and are used as adjuvants in cancer treatment. According to Rama and Prasad (6), high levels of α-tocopherol and γ-tocopherol in the blood decrease the metastasis risk of glioma in cancer patients. These substances play important roles in signal transduction and the regulation of gene expression (7).

Certain epidemiological and clinical studies have revealed that non-steroidal anti-inflammatory drugs (NSAIDs), which are used mainly in the treatment of autoimmune diseases, also have the potential to be used in cancer therapy (8–10).

Indomethacin is a strong NSAID derived from indolacetic acid. It has demonstrated antiproliferative effects on colon and breast cancers (11,12). Similar effects on glioma cells have also been reported (13). It also specifically increases the efficacy of two chemotherapeutics used in cancer treatment, namely doxorubicin and vincristine, in T98G human malignant glioma cells (14). Its antiproliferative and apoptosis-inducing effects are dependent on the treatment dose and time. The effects of other types of NSAIDs, such as ibuprofen, aspirin and naproxen, have also been investigated on glioma cell lines and some promising results have been obtained (13,15–17).

A common pharmacological property of NSAIDs is the inhibition of cyclooxygenases (COX1 and COX2). The expression of COX2 is known to increase markedly in cancer, and its activity is associated with the histological grade of the tumor and metastasis of the cancer (18–22). NSAIDs are able to prevent cancer progression by the inhibition of COXs (23,24). However, the antiproliferative effect may be independent from the inhibition of the enzyme (25). This type of drug may have certain roles in the induction of apoptosis, control of cell proliferation, invasion and inhibition of angiogenesis (26); however, the molecular mechanisms are not fully understood.

In the present study, the aim was to investigate the effects of α-TOS and indomethacin on oxidative stress parameters, by determining the intracellular oxidation level, molecular damage of proteins and lipids, and COX enzyme activity, in order to predict the possible effects of α-TOS in glioma treatment and/or prevention.

## Materials and methods

### Chemicals

α-tocopheryl succinate (α-TOS), indomethacin, phosphate-buffered saline (PBS) and 3-(4,5-dimethylthiazol-2-yl)-2,5-diphenyltetrazolium bromide (MTT) were purchased from Sigma Chemical Co. (St. Louis, MO, USA). Dulbecco’s modified Eagle’s medium (DMEM)/Nutrient Mixture F-12 HAM (F12 HAM) was purchased from Thermo Fischer Scientific Inc. (Waltham, MA, USA). Fetal bovine serum (FBS) was purchased from Gibco Life Technologies (Carlsbad, CA, USA). Antibiotic-antimicotic solution was purchased from Wisent Bioproducts (Quebec, Canada). Prestained protein molecular weight marker was obtained from Fermentas (Thermo Fisher Scientific).

### Maintenance of the cell line

Rat glioma cells (C6 line) were obtained from Cerrahpaşa Faculty of Medicine, Histology and Embryology Section (Istanbul, Turkey) and cultured in the laboratory. The cells were maintained in DMEM/F12 HAM containing 10% FBS, streptomycin (100 U/ml), penicillin (100 μg/ml) and amphotericin B (0.25 μg/ml) and were grown in an incubator (Heraeus, Thermo Fisher Scientific) at 37°C with 5% CO_2_. Stock solutions of α-TOS (25 mM in ethanol) and indomethacin (100 mM in DMSO) were diluted to the appropriate concentrations with DMEM/F12 HAM medium. Different concentrations of α-TOS (10, 25, 50, 100 and 200 μM) and indomethacin (50, 100, 200, 400, 500 and 600 μM) were tested in order to determine the CD_50_ value of each test material.

### Treatment of cells with test materials

The cultures were started in 96-well microplates with a cell number of 1×10^5^, with 200 μl cell suspension contained in each well. The culture media were removed from the plate at the end of the 24-h incubation period. The experimental groups were treated with α-TOS (10 μM), indomethacin (200 μM) and a combined mixture containing 10 μM α-TOS plus 200 μM indomethacin, whereas the control groups was incubated only with DMEM/F12 HAM, and the cells were maintained at 37°C and humidified with 5% CO_2_ for 48 h.

### Cytotoxicity tests

Cytotoxic concentrations were determined by a preliminary cytotoxicity test. The cell numbers were determined using the MTT assay (27) with minor modifications. Briefly, the culture media were removed at the end of 48-h incubation period. Then, 35 μl MTT solution prepared in PBS was added to each well and the microplates were incubated for 4 h. Following the incubation period, 200 μl DMSO was added to each well in order to solubilize the formazan crystals. After 15 min, the absorbance was measured at 570 and 690 nm (reference) using a microplate reader (μQuant, Bio-Tek Instruments, Inc., Winooski, VT, USA). The cell viability (%) of each group was calculated from the following formula: Cell viability (%) = (A_e_/A_c_) × 100, where A_e_ is the absorbance of the experimental group and A_c_ is the absorbance of the control group

### Intracellular oxidation level

The intracellular oxidation level was determined on the basis of spectrophotometric measurement of the fluorescent product formed by the oxidation of 2′,7′-dichlorofluorescein diacetate (DCF-DA) (28) When DCF-DA is normally introduced to the cell, it is reduced by the cleavage action of esterases. The reduced form (DCF-H) is then reoxidized to DCF by intracellular reactive oxygen species (ROS) and fluorescence is increased.

The cells (10^5^ cells) were firstly seeded onto a 96-well microplate. After incubation at 37°C under 5% CO_2_ for 24 h, the experimental groups were treated with α-TOS and/or indomethacin and incubated for a further 48 h. At the end of a total 72-h incubation period, the media were excluded and the wells were washed with PBS. A 200 μl addition of 5 μM DCF-DA solution was made to each well, and after 15 min the relative fluorescence was measured using a spectrofluorometer (FLx800; Bio-Tek), at the excitation and emission wavelengths 485 nm and 530 nm, respectively. Relative fluorescence (F) per minute was equalized based on the cell number in each well and the intracellular oxidation level was calculated using the following equation: Relative intracellular oxidation level (fluorescence % of control) = (Fe/Fc) × 100, where Fe is the fluorescence in the experimental group and Fc is the fluoresceence in the control group.

### Lipid peroxidation level

Thiobarbituric acid reactive substances (TBARS) as the end products of lipid peroxidation were detected by a spectrophotometric method (29). Control and experimental cells were transferred to a glass tube, containing 1 ml trichloroacetic acid (20%) and 0.8% (w/v) thiobarbituric acid, and boiled for 45 min. The samples were then cooled to room temperature and centrifuged at 3,000 × g for 5 min. The absorbance of the supernatant was measured at 535 nm. Malondialdehyde (MDA) with an extinction coefficient of 1.56×10^5^ M^−1^cm^−1^ at 535 nm, was used as a reference TBARS. The lipid peroxidation level in each sample was expressed as μM MDA equivalent/mg protein. The protein concentration of the samples was measured by bicinchoninic acid (BCA) assay as described previously (30).

### Protein carbonyls

OxyBlot kit (Chemicon; EMD Millipore, Billerica, MA, USA) was used to detect carbonyl groups in oxidatively modified proteins, as described by the manufacturer. Briefly, dinitrophenylhydrazine (DNPH) derivatization of 15 μg protein was carried out at room temperature for 15 min. Derivatized samples were then separated with 10% SDS-PAGE and transferred onto a nitrocellulose membrane (31). Red Ponceau S was used to determine the protein load following the transfer. The proteins on the membrane were probed with primary antibody, specific to DNP moieties of the proteins, followed by horseradish peroxidase-conjugated secondary antibody. Immunoblots were visualized using an Amersham ECL-Plus Western Blotting Detection system (GE Healthcare, Chalfont, UK) with an exposure time 2 min. A second gel containing duplicate samples was run and stained with Coomassie blue staining (32). Results were qualitatively evaluated using a prestained protein molecular weight marker.

### COX enzyme activity

COX enzyme activity was determined by measuring the MDA equivalent TBARS produced in the reaction mixture, as previously described (33).

The reaction mixture contained 100 mM Tris-HCl, pH 8.0, 5 mM reduced glutathione (GSH), 5 μM hemoglobin and soluble proteins extracted from the cells. Arachidonic acid at a final concentration of 0.5 mM was then added to the reaction mixture. Following incubation at 27°C for 1 min, the reaction was stopped by the addition of 0.2 ml 100% (w/v) trichloroacetic acid (prepared in 1 M HCl) and keeping the mixture in a boiling water bath for 20 min. The samples were centrifuged (1,000 × g, 5 min) and the absorbance of the supernatant was measured at 532 nm.

The COX activity was expressed in units of nmol MDA equivalent/min, using the molar extinction coefficient of MDA (1.56×10^5^ M^−1^cm^−1^).

### Statistical analyses

All experiments were carried out at least three times in triplicate and data were analyzed by unpaired analysis of variance (ANOVA) using the Graph Pad Prism software package, version 5.0 (GraphPad Software, Inc., La Jolla, CA, USA).

## Results

### Cytotoxicity

**Figure 1:**
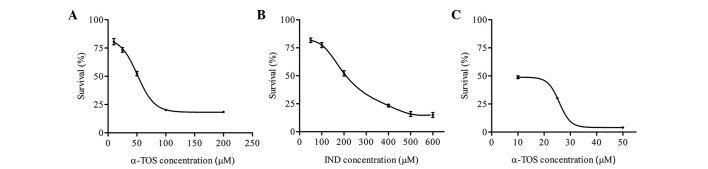
Effect of (A) α-TOS, (B) IND and (C) α-TOS in combination with 200 μM IND on cell survival in the C6 glioma cell line. Experiments were performed in triplicate. The vertical bars on the points on the graph show standard deviation values. The consistency between groups was determined by one-way analysis of variance (P<0.001; R^2^, 0.996). α-TOS, α-tocopheryl succinate; IND, indomethacin.

The CD_50_ values were found to be 50 μM for α-TOS and 200 μM for indomethacin (Fig. 1A and B). When a combination containing 50 μM α-TOS and 200 μM indomethacin was applied, the viability of the cells was only 4% (data not shown). Thus, different concentrations of α-TOS were added to 200 μM IND and the cell viability was evaluated. A combination of 10 μM α-TOS and 200 μM indomethacin was used in further experiments, as this combination caused ~50% survival of the cells.

### Oxidative stress parameters

**Figure 2:**
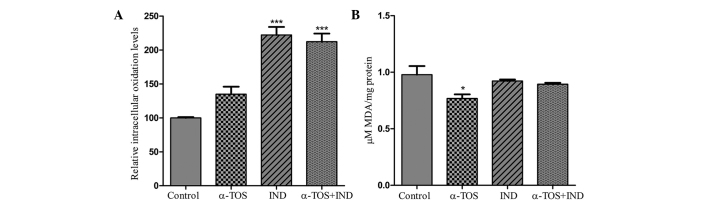
Oxidative stress parameters in C6 glioma cells. (A) ROS levels and (B) lipid peroxidation (TBARS levels). Experiments were performed in triplicate. The vertical bars above the columns show standard deviation values. The consistency between groups was determined by one-way analysis of variance ^*^P<0.05, ^***^P<0.001 compared with the control group. ROS, reactive oxygen species; TBARS, thiobarbituric acid reactive substances; α-TOS, α-tocopheryl succinate; IND, indomethacin; MDA, malondialdehyde.

The levels of ROS were increased by 34.6, 122.2 and 112.5%, by the treatment of the cells with α-TOS, IND and α-TOS + IND, respectively (Fig. 2A). Treatment with 200 μM indomethacin alone or in combination with α-TOS (10 μM) induced intracellular oxidation by at least three-fold more than treatment with α-TOS alone.

The level of TBARS generated as a result of lipid peroxidation was 0.978±0.133 μM MDA/mg protein in control cells. Following treatment with 10 μM α-TOS, lipid peroxidation was found to decrease to 0.768±0.0626 μM MDA/mg protein (Fig. 2B), while in the IND and α-TOS + IND groups, the TBARS level was almost the same as that in the control group, at IND 0.922±0.0241 and 0.894±0.0215 μM MDA/mg protein, respectively.

**Figure 3:**
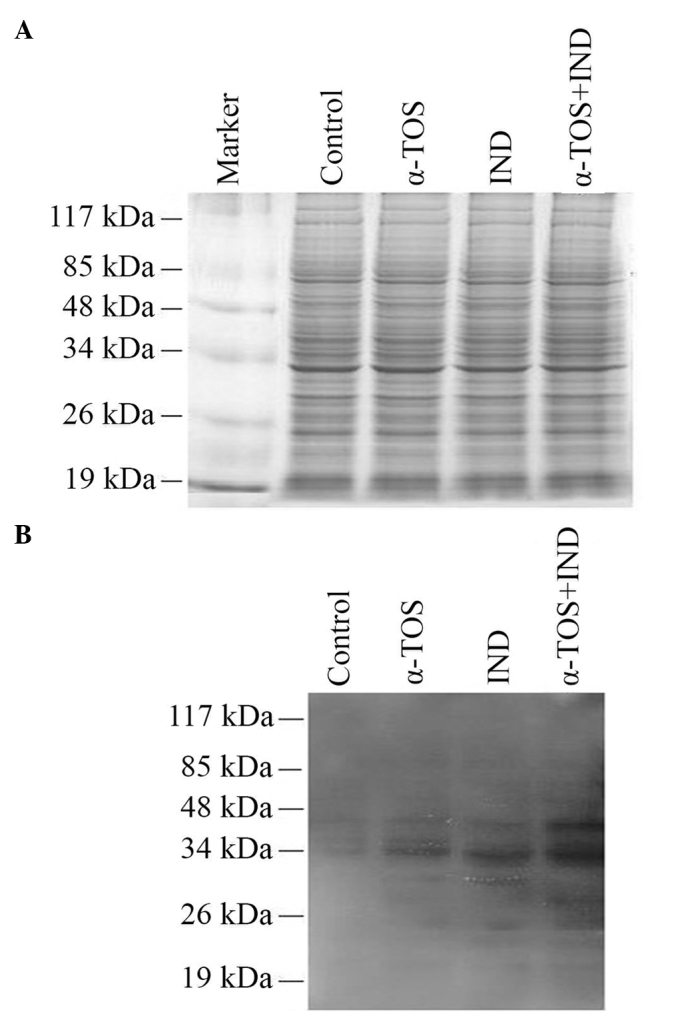
(A) Protein profiles in a denaturated gel stained with Coomassie blue. (B) Carbonylated proteins on a X-ray film. Protein mixture containing lysozyme (19 kDa), β-lactoglobulin (26 kDa), carbonic anhydrase (34 kDa), ovalbumin (48 kDa), bovine serum albumin (85 kDa) and β-galactosidase (117 kDa) was used as a marker. α-TOS, α-tocopheryl succinate; IND, indomethacin.

Protein damage was evaluated by the analysis of carbonylated proteins on a nitrocellulose membrane by western blotting. All three treatments induced protein carbonylation (Fig. 3B). At least 10 polypeptides were detected as carbonylated in all samples. The most intense carbonyl bands were observed for the α-TOS + IND group.

### COX enzyme activity

The COX enzyme activities were measured, as it is known that IND acts as an inhibitor of this enzyme. IND treatment lowered the enzyme activity by 39.17%. Notably, 10 μM α-TOS also decreased COX activity by 22.7%. Thus α-TOS appeared to inhibit COX (Table I). The combination of α-TOS + IND reduced the COX activity by 46.39% compared with that in the control.

## Discussion

The main problems in the treatment of brain cancers by chemotherapy, radiotherapy and surgery are the side-effects, such as pain, vomiting and damage to healthy cells and tissues that results in several complications. Due to the feelings of sadness, fear, anxiety and anger that patients experience, and as a result of potential for mortality, patients often turn to complementary and alternative therapies (2). NSAIDs have been found to inhibit the progression and invasion of various glioma tumors (17,34) Indomethacin is an NSAID that is able to pass through the blood-brain barrier and inhibit COX enzymes irreversibly (35). Thus, indomethacin has been proposed as a potential drug for use in glioma therapy (14).

The administration of antineoplastic agents during cancer chemotherapy results in a much greater degree of oxidative stress than is induced by the cancer itself (36). The high level of oxidative stress during chemotherapy may be overcome by the body’s oxidative defense systems, using antioxidants specialized mainly to reduce lipid peroxidation. In addition, complementary nutritional therapy with antioxidants such as vitamin E (mixed tocopherols and tocotrienols), β-carotene (natural mixed carotenoids), vitamin C (ascorbic acid) and vitamin A (retinoic acid) during chemotherapy may inhibit the effect of oxidative stress on healthy cells and tissues, and the development of multidrug resistance in cancer cells (1). Antioxidant supplements are also a common choice for patients who try complementary and alternative methods in addition to conventional therapies. While it is accepted that antioxidants are useful in the reduction of the adverse effects of chemotherapy, the prevailing opinion is that they may reduce the effectiveness of chemotherapy (37,38).

Vitamin E is a remarkable supplement for cancer therapy (2), due to its potential effect in the reduction of oxidative stress during chemotherapy (39). However, its effects on drug metabolism as well as on the stress response of cancer cells are not yet fully documented.

In the present study, the aim was to elucidate the effects of α-TOS, a water-soluble vitamin E derivative, on glioma cells treated with indomethacin, a potential chemotherapeutic agent. It was found that 50% of C6 glioma cells survived in the presence of 50 μM α-TOS or 200 μM indomethacin in the medium. α-TOS was also implemented in combination with indomethacin. In the combination, the concentration of the components was selected as 10 μM for α-TOS and 200 μM for indomethacin. The antiproliferative effect of NSAIDs may also be explained by the direct inhibition of COX-2 (40). However, this mechanism is not eligible for C6 glioma cells, since these cells are not able to express COX-2 (41). Therefore, the data obtained in the present study may be associated with COX-1 inhibition by indomethacin.

At the end of the 48-h implementation period, the oxidative stress as well as molecular damage that occurred in the cells were analyzed by measuring the intracellular oxidation level, lipid peroxidation and protein carbonyls. In addition, COX activity was measured in order to determine the degree of inhibition of this enzyme by α-TOS, alone or along with indomethacin.

In this study, all treatments induced intracellular ROS production (Fig. 2A). Although α-TOS has been reported to be a strong antioxidant (42), a pro-oxidant effect of α-TOS was exhibited for the concentration that was used in this study. Similar results have been reported in murine melanoma, malignant mesothelioma and breast cancer cells (22,43,44). Indomethacin and α-TOS induced ROS production in the reaction system of the present study. ROS production by indomethacin is well documented in gastric mucosal cells (45,46); however, to the best of our knowledge, the present study is the first to report on indomethacin-induced ROS production in C6 glioma cells. Although, ROS generation was detected in the cells treated with α-TOS and/or indomethacin, lipid peroxidation was not induced. Moreover, lipid damage was reduced in the α-TOS group compared with that in the control (Fig. 2B). Similar results were obtained in a previous study in which a murine melanoma cell line was treated with 0.15 μM indomethacin plus 1–10 μg/ml α-TOS (43). This protective effect may result from an inhibitory effect of α-TOS on lipid peroxidation (7).

The oxidative modifications of proteins (carbonylation) may lead to a loss of specific function (47,48) and contribute to neuronal cell injury and death. Accordingly, the levels of protein carbonyls following α-TOS and/or indomethacin treatment were also measured in the present study. The induction of protein carbonylation by all treatments indicates that ROS produced by α-TOS and indomethacin interacted with cellular proteins.

The present study briefly demonstrates that α-TOS and/or indomethacin induce protein damage, but inhibit lipid peroxidation in C6 glioma cells. In particular, indomethacin induces the generation of ROS and causes molecular damage while exhibiting its effects. The results of the study indicate that α-TOS does not have a negative effect on the action of indomethacin. Further studies are required with animal models to observe the efficacy of α-TOS more clearly. These results, in combination with the documented literature, may be useful in the development of new glioma therapies.

## Figures and Tables

**Table I:** Cyclooxgenase activity in C6 glioma cells.

Group	Cyclooxgenase activity (nmol MDA equivalent/min)
Control	97±6.76
α-TOS	75±4.82
IND	59±3.56
α-TOS + IND	52±4.16

Values are mean ± standard deviation. α-TOS, α-tocopheryl succinate; IND, indomethacin; MDA, malondialdehyde.
